# DNA-terminus-dependent transcription by T7 RNA polymerase and its C-helix mutants

**DOI:** 10.1093/nar/gkae593

**Published:** 2024-07-09

**Authors:** Bingbing Yu, Yifan Chen, Yan Yan, Xueling Lu, Bin Zhu

**Affiliations:** Key Laboratory of Molecular Biophysics, Ministry of Education, College of Life Science and Technology, Huazhong University of Science and Technology, Wuhan, Hubei 430074, China; Key Laboratory of Molecular Biophysics, Ministry of Education, College of Life Science and Technology, Huazhong University of Science and Technology, Wuhan, Hubei 430074, China; Key Laboratory of Molecular Biophysics, Ministry of Education, College of Life Science and Technology, Huazhong University of Science and Technology, Wuhan, Hubei 430074, China; Key Laboratory of Molecular Biophysics, Ministry of Education, College of Life Science and Technology, Huazhong University of Science and Technology, Wuhan, Hubei 430074, China; Key Laboratory of Molecular Biophysics, Ministry of Education, College of Life Science and Technology, Huazhong University of Science and Technology, Wuhan, Hubei 430074, China

## Abstract

The remarkable success of messenger RNA (mRNA)-based vaccines has underscored their potential as a novel biotechnology platform for vaccine development and therapeutic protein delivery. However, the single-subunit RNA polymerase from bacteriophage T7 widely used for *in vitro* transcription is well known to generate double-stranded RNA (dsRNA) by-products that strongly stimulate the mammalian innate immune response. The dsRNA was reported to be originated from self-templated RNA extension or promoter-independent transcription. Here, we identified that the primary source of the full-length dsRNA during *in vitro* transcription is the DNA-terminus-initiated transcription by T7 RNA polymerase. Guanosines or cytosines at the end of DNA templates enhance the DNA-terminus-initiated transcription. Moreover, we found that aromatic residues located at position 47 in the C-helix lead to a significant reduction in the production of full-length dsRNA. As a result, the mRNA synthesized using the T7 RNA polymerase G47W mutant exhibits higher expression efficiency and lower immunogenicity compared to the mRNA produced using the wild-type T7 RNA polymerase.

## Graphical Abstract

**Graphical Abstract:**
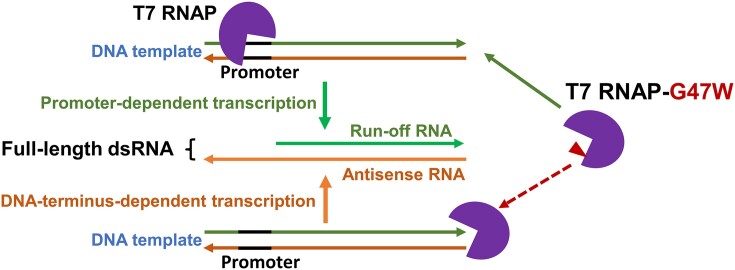

## Introduction

The extraordinary success of messenger RNA (mRNA)-based vaccines against the COVID-19 pandemic highlighted the potential of mRNA-based biotechnology for vaccine development and therapeutic protein delivery (1–4). As an emerging class of medicines, mRNA-based therapeutics are considered safer, more efficient and more economical compared to DNA-based therapeutics and conventional protein/peptide drugs (5,6). The only available method to produce large amounts of designed mRNA is the *in vitro* transcription (IVT) carried out by single-subunit RNA polymerases (ssRNAPs). However, the most popular ssRNAP, from bacteriophage T7, is known to generate undesired double-stranded RNA (dsRNA) by-products that can stimulate the mammalian innate immune system (7,8). The dsRNA by-products of T7 RNAP are mainly generated by self-template extension (9–13) or promoter-independent transcription (14,15). For therapeutic applications that require as much as a 1000-fold higher level of protein than vaccines to reach the therapeutic threshold, it is necessary to reduce the immunostimulatory effect of the dsRNA to a safe level (1–5).

Previous studies demonstrated that incorporation of modified nucleotides such as *N*^1^-methylpseudouridine could reduce the immunogenicity of *in vitro* transcribed RNA (16–20). Certain modified nucleotides inhibit the generation of dsRNA by-products in T7-RNAP-based IVT (15,21). Other efforts to reduce the production of dsRNA by-products include applying high-salt transcription conditions with tight-binding promoter variants (22), co-tethering promoter DNA and T7 RNAP on magnetic beads (23), adding competing 3′-capture DNA (24) or chaotropic agents (2), and lowering the Mg^2+^ concentration (15,21). In addition, post-transcriptional purification techniques such as reverse-phase high-pressure liquid chromatography (18,25) or cellulose chromatography (26) can also reduce dsRNA contaminants. Nevertheless, the above methods would increase manufacturing costs, decrease yield and/or introduce new contaminants.

Recent studies have focused on ssRNAP, the core component of IVT, to reduce dsRNA production. Lu *et al.* and Xia *et al.* reported that two novel ssRNAPs, from *Klebsiella* phage KP34 (27) and *Pseudomonas* phage VSW-3 (28,29), respectively, produce minimal self-templated dsRNA. Wu *et al.* reported that thermostable T7 RNAP mutants are able to synthesize functional mRNA with reduced immunogenicity at a high temperature (50°C) (30). Wu *et al.* reported that a single mutation S43Y attenuated RNA-dependent RNAP activity of T7 RNAP by weakening the RNA rebinding (31). Dousis *et al.* reported that an engineered T7 RNAP containing mutations G47A and 884G increased the 3′ homogeneity of transcribed RNA from 6–12% to >90% (32).

dsRNA contaminants in IVT products include short dsRNA and long dsRNA similar in length to the desired single-stranded RNA (ssRNA) transcript (referred to as the full-length dsRNA). Both types of dsRNA may trigger cellular immune responses: dsRNA regions as short as 40 bp are sensed by dsRNA receptors such as Toll-like receptor 3 (33), while dsRNA regions larger than 500 bp are sensed by melanoma differentiation-associated protein 5 in mammalian cells (7,34). Previous study demonstrated that the full-length dsRNA is derived from the promoter-independent DNA-terminus-initiated transcription of T7 RNAP (15). In this work, we focused on the full-length dsRNA and solutions to reduce its production. We identified the initiation sites of the promoter-independent transcription of T7 RNAP and found that the guanosines and cytosines at the end of DNA templates enhance the promoter-independent transcription. We carried out a tyrosine screen on residues 42–48 in the C-helix of T7 RNAP, which is potentially involved in the interaction between the enzyme and the DNA template (31,32,35,36), and found that substitutions of G47 with aromatic amino acids attenuate the promoter-independent transcription significantly. Among these T7 RNAP mutants, G47W was found to produce the least full-length dsRNA.

## Materials and methods

### Protein expression and purification

DNA fragments encoding T7 RNAP variants were inserted into the pQE82L vector and DNA fragments encoding wild-type (WT) SP6 RNAP were inserted into the pET28b vector with N-terminal His tags. The vectors were transformed into *Escherichia coli* BL21(DE3), and cells were cultured in 1 l LB medium containing 100 mg/ml ampicillin (for T7-RNAP-pQE82L vector) or 50 mg/ml kanamycin (for SP6-RNAP-pET28b vector) at 37°C until the OD_600_ reached 1.2. Overexpression of RNAPs was induced by addition of 0.5 mM isopropyl β-D-1-thiogalactopyranoside and continuous incubation at 16°C for 16 h. The cells were collected, resuspended in buffer (50 mM Tris–HCl, pH 7.5, 300 mM NaCl) and lysed by ultrasonication. The supernatants were filtered through the 0.2 μm filters and then loaded into the Ni-NTA-agarose columns (Qiagen) equilibrated with wash buffer containing 50 mM Tris-HCl, pH 7.5, and 300 mM NaCl. After the supernatants flowing through, the Ni-NTA-agarose columns were eluted with wash buffer containing gradient imidazole (50 mM Tris-HCl, pH 7.5, 300 mM NaCl, 20 mM, 40 mM, 60 mM, 80 mM or 120 mM imidazole) and each 6 ml eluents were collected. All the eluents were analyzed by SDS-PAGE and the fractions containing RNAP with a purity over 80% were collected and concentrated for next steps. Then the RNAPs were purified using HiTrap Q HP columns (Cytiva) with a start buffer (20 mM Tris–HCl, pH 8.0) and a gradient elution buffer (20 mM Tris–HCl, pH 8.0, 0–1 M NaCl) and a flow rate of 1 ml/min. Eluted fractions were analyzed by SDS-PAGE and the fractions containing RNAP with a purity over 90% were collected and concentrated. Finally, the concentrated proteins were dialyzed two times against dialysis buffer [100 mM NaCl, 50 mM Tris–HCl, pH 7.5, 1 mM dithiothreitol (DTT), 0.1 mM ethylenediaminetetraacetic acid, 50% glycerol, 0.1% Triton X-100]. VSW-3, KP34 and Syn5 RNAPs were purified as described previously (27–29,37,38). Protein purity was analyzed by SDS-PAGE and shown as Supplementary Figure S1.

### Transcription assays

Sequences of the DNA templates and primers are shown in Supplementary Table S1. All the DNA templates were prepared by polymerase chain reaction (PCR), unless otherwise noted. IVT reactions by T7, KP34 (27), SP6 (39) or VSW-3 RNAP (28) contained 40 mM Tris–HCl (pH 8.0), 15 mM MgCl_2_, 2 mM spermidine, 5 mM DTT, 0.2 μM inorganic pyrophosphatase, 1.5 U/μl RNase inhibitor, 4 mM each of ATP, CTP, GTP and UTP, 30 ng/μl DNA templates and 0.2 μM RNAP. The reaction mixtures were incubated at 37°C for 2 h (unless otherwise indicated) or at 25°C for 16 h (only for VSW-3 RNAP). IVT reactions by Syn5 RNAP containing 40 mM Tris–HCl (pH 8.0), 15 mM MgCl_2_, 2 mM spermidine, 5 mM DTT, 0.2 μM inorganic pyrophosphatase, 1.5 U/μl RNase inhibitor, 4 mM each of ATP, CTP, GTP and UTP, 30 ng/μl DNA templates and 2 μM RNAP were incubated at 30°C for 4 h (37). After incubation, 1 unit of DNase I was added to the reaction mixtures to remove DNA templates. In some assays, the DNase I treatment was omitted to show the DNA templates along with the transcripts. All the reactions were stopped by mixing with the RNA loading dye (47.5% formamide, 0.01% SDS, 0.01% bromophenol blue, 0.005% xylene cyanol and 40 mM EDTA) before electrophoresis. The IVT products were purified with Monarch RNA purification kits (New England Biolabs), and the purified RNA was quantified using a nanophotometer (Implen).

To show the abortive products during the transition from transcription initiation to elongation, 0.32 mM fluorescently labeled dinucleotide 6-FAM-GG (Takara) was added into the IVT reactions containing 40 mM Tris–HCl (pH 8.0), 15 mM MgCl_2_, 2 mM spermidine, 5 mM DTT, 0.2 μM inorganic pyrophosphatase, 1.5 U/μl RNase inhibitor, 4 mM each of ATP, CTP, GTP and UTP, 1 μM DNA templates and 0.2 μM RNAP. One micromolar fluorescently tagged RNA was heated at 90°C for 15 min in 50 mM NaOH, and then was used as an RNA ladder to mark 1–39 nt RNA. The gels were imaged and analyzed using a ChemiScope 6000 Imaging System (Clinx).

### RNase III/RNase I_f_ digestion

For RNase digestion, 600 ng GFP RNA was incubated with 1, 2, 4 or 8 μl RNase III (1:1000 diluted, New England Biolabs) or RNase I_f_ (1:100 diluted, New England Biolabs) in buffer 3 (100 mM NaCl, 50 mM Tris–HCl, pH 7.9, 10 mM MgCl_2_ and 1 mM DTT, New England Biolabs) or in 50 mM NaCl, 50 mM Tris–HCl (pH 7.5), 20 mM MnCl_2_ and 1 mM DTT at 37°C for 30 min. Reactions were terminated by heat inactivation at 85°C for 5 min.

### Native gel electrophoresis

To visually distinguish ssRNA and dsRNA, IVT transcripts were analyzed by 6% TBE polyacrylamide gel electrophoresis and stained by acridine orange (Sigma–Aldrich). Fluorescence gel images were obtained by a ChemiScope 6000 Imaging System (Clinx). For ssRNA-specific image, the 473-nm filter was used for excitation and the 792-nm filter was used for emission. For dsRNA-specific image, the 532-nm filter was used for excitation and the 554-nm filter was used for emission. The fluorescence images were superimposed.

### Dot blot

Various concentrations of IVT transcripts were dropped onto an Immobilon TM-Ny+ Membrane (Millipore), which was dried, blocked with 5% non-fat dry milk in TBS-T buffer (50 mM Tris–HCl, pH 7.4, 150 mM NaCl and 0.05% Tween 20) and incubated with dsRNA-specific J2 mAb (Scicons) for 30 min at 25°C. The membrane was washed three times with TBS-T buffer and incubated with hydrogen peroxidase-conjugated donkey anti-mouse Ig (1:2000 diluted, Jackson Immunology). After washing the membrane three times, chemiluminescence detection was performed using the ECL™ Enhanced Pico Light Chemiluminescence Kit (EpiZyme) and a ChemiScope 6000 Imaging System (Clinx).

### RNA 5′RACE and 3′RACE

A 200-nt RNA linker was prepared by IVT and its triphosphate group was converted to a monophosphate group by RppH (New England Biolabs) for 3′RACE. Then, the GFP RNA was linked to the treated linker with T4 RNA ligase 1 (New England Biolabs). The obtained RNA was purified with the Monarch RNA purification kit (New England Biolabs), and reverse transcription was performed with a specific primer using MagicScript thermotolerant reverse transcriptase (Magigen). Then, the complementary DNA (cDNA) was amplified by PCR with a pair of specific primers and purified with the AxyPrep PCR Cleanup Kit (Axygen). The cDNA was inserted into the plasmid pUC18 and subjected to Sanger sequencing (GeneCreate). For 5′RACE, the GFP RNA was treated with RppH and linked to the 200-nt RNA linker, following the same steps as those for 3′RACE. All primers used are listed in Supplementary Table S1.

### DNA binding

5′-6-FAM fluorescently tagged DNA fragments (Genscript, sequences are shown in Supplementary Table S1) with or without T7 promoter were annealed in 10 mM Tris–HCl (pH 7.5) and 5 mM NaCl. Then, 10 nM DNA hairpins were incubated with various concentrations (0, 25, 50, 100, 200, 400 and 800 nM) of WT T7 RNAP or its G47W or E45K mutant in 40 mM Tris–HCl (pH 8.0), 15 mM MgCl_2_, 2 mM spermidine and 5 mM DTT at 37°C for 20 min. The mixtures were mixed with 5 μl loading dye (0.01% SDS, 0.01% bromophenol blue, 0.005% xylene cyanol) and separated by 10% TBE PAGE at 100 V for 70 min. The gels were imaged using a ChemiScope 6000 Imaging System (Clinx) and images were analyzed by ImageJ.

### Quantitative analysis

The gray value of gel bands and immunoblot dots was quantified with ImageJ software, and the diagrams were generated by Prism.

### mRNA synthesis

First, 3.2 mM CleanCap AG (3′ OMe) (Trilink) was added to the IVT reaction by T7 RNAP or T7 RNAP-G47W to obtain 5′-capped GFP RNA. The capped RNA was purified with the Monarch RNA purification kit, and a poly(A) tail was added to the capped RNA by *E. coli* poly(A) polymerase (New England Biolabs). The final GFP mRNA was purified with the Monarch RNA purification kit.

### Cell culture and flow cytometry

HEK293T cells were cultured in 24-well plates (NEST) in Dulbecco’s modified Eagle medium (Gibco) supplemented with 10% fetal calf serum (Gibco), 1% penicillin/streptomycin (Thermo Fisher Scientific) and 2.5 mg/ml plasmocin prophylactic (Invivogen). At ∼80% confluence, the cells were transfected with 500 ng GFP mRNA encapsulated in Lipofectamine 2000 (Thermo Fisher Scientific) as per the manufacturer’s protocol. GFP expression levels were recorded by fluorescence microscopy at 4, 8 and 20 h after transfection. After 20 h, fluorescence intensity was quantified by flow cytometry using SH800S (Sony).

### IFN-β detection

HEK293T cells were cultured in six-well plates as described above. At ∼80% confluence, the cells were transfected with 2000 ng GFP mRNA encapsulated in Lipofectamine 2000 (Thermo Fisher Scientific) as per the manufacturer’s protocol. After 20 h, the cells were collected, lysed and centrifuged, and the IFN-β in the supernatant was detected using the Human IFN-β (Interferon Beta) ELISA Kit (Elabscience) as per the manufacturer’s protocol.

## Results

### T7 RNAP produces full-length dsRNA by-products in IVT

**Figure 1.**
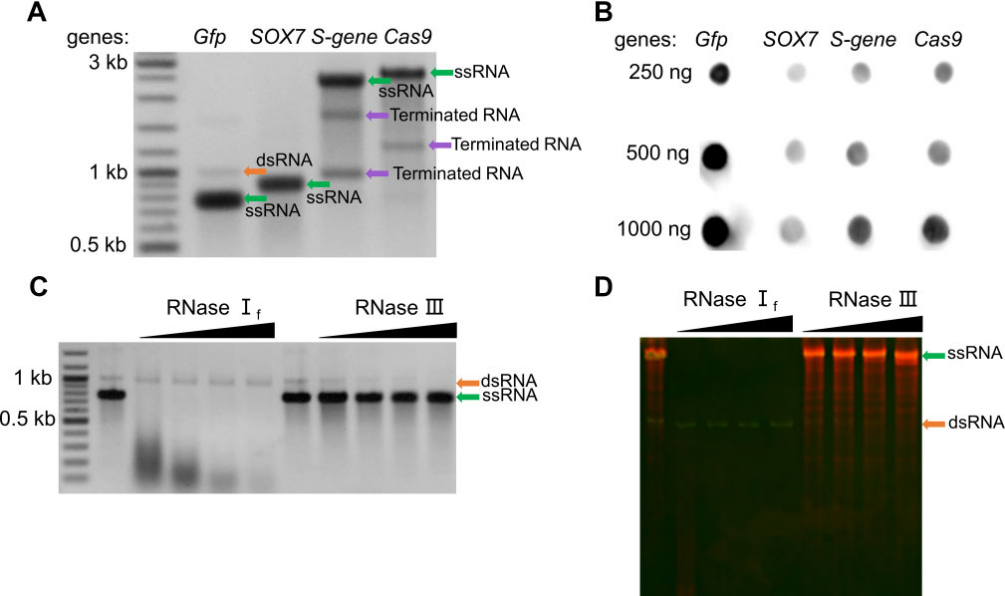
Full-length dsRNA in the IVT products of T7 RNAP. (**A**) 1.5% agarose gel electrophoresis analysis and (**B**) dot blot analysis of four genes transcribed by T7 RNAP. (**C**) 1.5% agarose gel electrophoresis analysis of GFP transcripts treated with RNase I_f_ (1/40, 1/20, 1/10 and 1/5 units/μl) and RNase III (1/10 000, 1/5000, 1/2500 and 1/1250 units/μl). (**D**) 6% native PAGE analysis of GFP transcripts treated with RNases as in panel (C). The gel bands were visualized by acridine orange staining. Gel bands corresponding to ssRNA, dsRNA and terminated RNA are indicated by arrows, respectively, in all gels.

Four common DNA templates (*gfp*, *sox7*, the *S-gene* from SARS-CoV-2 and *cas9*) were transcribed by T7 RNAP. The IVT products were analyzed by native agarose gel electrophoresis (Figure 1A) and dot blot analysis (Figure 1B). The dot blot assay detected dsRNA by-products in all the transcripts, with the highest dsRNA content in GFP transcripts (Figure 1B). Consistently, we observed an obvious band above the gel band corresponding to the desired GFP ssRNA (Figure 1A). The position of the upper band indicates a by-product corresponding to the full-length GFP dsRNA. To confirm the identity of this by-product, we digested the GFP transcripts with RNase I_f_ and RNase III, which specifically degrade ssRNA and dsRNA, respectively. Then, the RNase-treated transcripts were analyzed by native agarose gel electrophoresis or PAGE, followed by ethidium bromide or acridine orange dyeing, as previously reported (15). The upper band was sensitive to RNase III but not to RNase I_f_ (Figure 1C), confirming that it represents dsRNA. As expected for the full-length dsRNA, it migrated faster than the single-stranded GFP RNA in the native PAGE (Figure 1D). Acridine orange staining further distinguished the GFP ssRNA from the full-length dsRNA (Figure 1D). To reveal the mechanism of full-length dsRNA formation, we focused on the GFP transcripts with the most significant full-length dsRNA content for following investigations.

### T7 RNAP initiates transcription from DNA terminus without promoter

**Figure 2.**
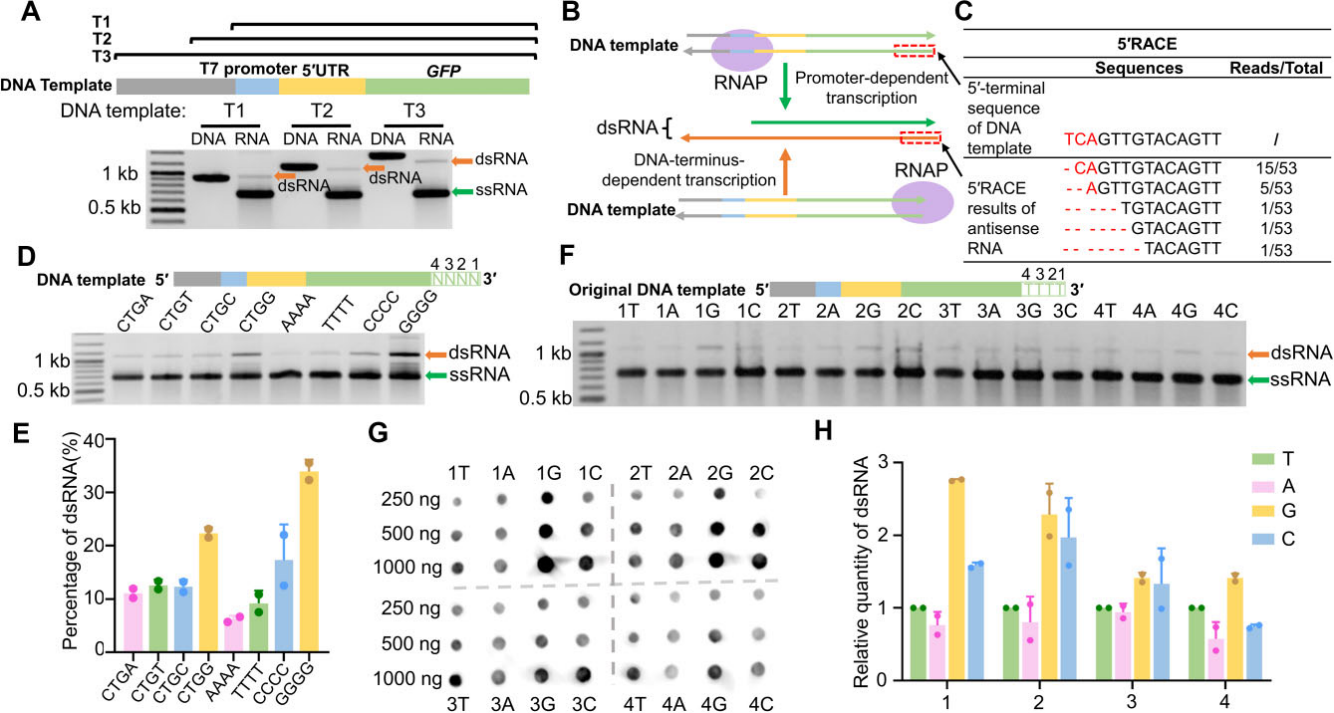
DNA-terminus-initiated transcription by T7 RNAP. (**A**) 1.5% agarose gel electrophoresis analysis of the IVT reactions by T7 RNAP on DNA templates with 5′ extensions. The noncoding sequences upstream of the T7 promoter of templates T1, T2 and T3 were 53, 290 and 583 bp in size, respectively. DNA indicates the DNA templates and RNA indicates the transcripts. (**B**) Schematic showing the formation of full-length dsRNA in IVT. (**C**) 5′RACE results of antisense RNA. Corresponding positions of listed DNA and RNA sequences are indicated in the dashed boxes in panel (B). (**D**) Agarose gel electrophoresis analysis of the IVT products of T7 RNAP on DNA templates with various termini. The last 4-nt sequences of DNA templates are indicated on top of the gel. (**E**) Quantification of the full-length dsRNA shown in panel (D). The gray values of the gel bands corresponding to the runoff ssRNA and the full-length dsRNA were measured, and the percentage of full-length dsRNA was calculated as the ratio between the full-length dsRNA and the sum of the runoff ssRNA and the full-length dsRNA. (**F**) Agarose gel electrophoresis analysis of the IVT products of T7 RNAP on DNA templates with various termini. The original DNA template ends with four Ts. Each of these four Ts was substituted with A, G or C, and the production of full-length dsRNA on these templates was analyzed. (**G**) Dot blot analysis of transcripts shown in panel (F), demonstrating their full-length dsRNA contents. (**H**) Quantification of the full-length dsRNA shown in panel (G). dsRNA content was normalized to that obtained using the original DNA template (four Ts). DNA templates, ssRNA and dsRNA are indicated by arrows, respectively, in all gels.

Previous studies reported that the large dsRNA by-products of T7 RNAP were mainly generated by self-templated RNA extension (9–13) or promoter-independent transcription (15,21). To reveal the origin of the full-length dsRNA in GFP transcripts, we extended the *gfp* DNA templates with 53-, 290- or 583-bp noncoding sequences at the 5′ termini, upstream of the T7 promoter (Figure 2A). These extensions do not affect the desired GFP ssRNA initiated from the promoter, so the size of the full-length dsRNA would not change if it originated from the self-templated RNA extension as described previously (9–13). However, if T7 RNAP initiates transcription at the 3′-termini of the DNA template, the antisense GFP transcript would be extended following the extension of the antisense DNA, and the size of the full-length dsRNA annealed by the GFP transcript (initiated at the promoter) and the antisense GFP transcript (initiated at the 3′ DNA terminus) would also increase. As shown in Figure 2A, the gel bands corresponding to the GFP ssRNA had the same mobility, despite the template 5′ extension. However, the gel mobilities of the upper bands corresponding to the full-length dsRNA were lower following the template 5′ extensions, confirming that the full-length dsRNA in GFP transcripts originated from promoter-free DNA-terminus-initiated transcription (Figure 2B) but not from RNA-templated self-extension. We also performed IVT on GFP DNA lacking T7 promoter (Supplementary Figure S2) and the results showed that T7 RNAP still produces the full-length dsRNA on such DNA template, confirming the promoter-independent transcription by T7 RNAP. To further characterize the antisense transcript, we performed 3′RACE and 5′RACE analyses on the antisense RNA from the GFP DNA template with a 290-bp 5′ extension. The results of our 5′RACE analysis showed that most of the antisense RNA initiated from the second base at the nonpromoter end of the DNA template (Figure 2C). 3′RACE analysis showed that the 3′ sequence of the antisense RNA matches the 5′ sequence of the antisense strand of the template DNA, confirming the runoff termination of the antisense RNA (Supplementary Table S2).

Previous studies (14,15) have demonstrated that the terminal structures of DNA templates influence the production of dsRNA by T7 RNAP, with the 3′ protruding DNA ends leading to more dsRNA production. The result that the production of full-length dsRNA (Figure 1A and B) varied among various DNA templates (all produced by PCR with blunt ends) indicates that the terminal sequences of DNA templates affect the DNA-terminus-initiated transcription by T7 RNAP. To clarify such influence, we prepared eight blunt-ended DNA templates with various 4-bp terminal sequences by PCR and examined their transcripts generated by T7 RNAP (Figure 2D). We calculated the proportion of dsRNA and found that DNA templates with four consecutive Gs at the ends yielded the most full-length dsRNA (Figure 2E). In contrast, DNA templates with four As or Ts at the ends yielded the least dsRNA. These results are consistent with a previous report showing that adding poly(dA) to the 3′ end of DNA templates reduces dsRNA generation in T7 RNAP IVT (30).

We further tested the effect of single variations in the 4-bp (5′-TTTT-3′) template terminal sequence on the production of full-length dsRNA. Each of the four terminal Ts was replaced by G, C or A. The IVT products were analyzed by agarose gel electrophoresis (Figure 2F) and dot blot analysis (Figure 2G). Then, we quantified the dsRNA from these templates based on Figure 2G and compared them to that from the original template (Figure 2H). Results showed that the last two bases have the most significant impact on the generation of full-length dsRNA; a single guanosine or cytidine in the terminal 2-nt region increases the yield of full-length dsRNA.

### DNA-terminus-dependent transcription is not common for bacteriophage ssRNAPs

**Figure 3.**
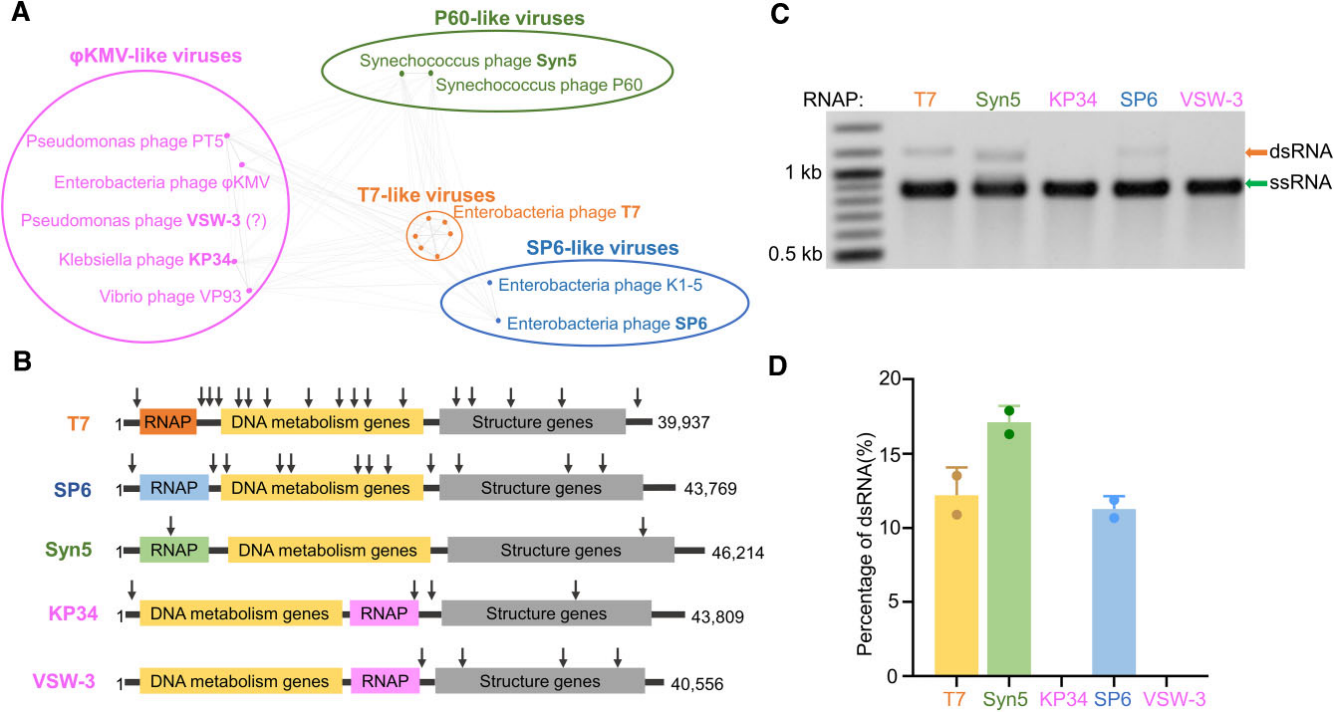
Representative ssRNAPs and their production of full-length dsRNA in IVT. (**A**) Schematic showing *Autographivirinae* clusters based on genome sequence comparison [an inaccurately modified version of Figure 3 in (40)]. (**B**) Schematic illustration of the genome organization of phages T7, SP6, Syn5, KP34 and VSW-3. The approximate positions of T7, SP6 and Syn5 promoters are indicated by downward arrows. Genome sizes are also shown. (**C**) Agarose gel electrophoresis analysis of the GFP transcripts synthesized by representative ssRNAPs in IVT. (**D**) Quantification of the results shown in panel (C). The gray values of the gel bands corresponding to the runoff ssRNA and the full-length dsRNA were measured, and the percentage of the full-length dsRNA was calculated as the ratio between the full-length dsRNA and the sum of the runoff ssRNA and the full-length dsRNA. Gel bands corresponding to ssRNA and dsRNA are indicated by arrows, respectively.

Bacteriophages encoding ssRNAPs belong to the *Autographivirinae*, a subfamily of the *Podoviridae*, which were classified into four distinct clusters corresponding to phiKMV-, P60-, SP6- and T7-like viruses (40) (Figure 3A). We have characterized Syn5 (38) and KP34 (27) RNAPs as the representative ssRNAPs of the P60-like and phiKMV-like bacteriophages distantly related to T7, respectively. Bacteriophage VSW-3, which encodes another recently characterized ssRNAP (28), most likely also belongs to the cluster of phiKMV-like viruses based on its genomic organization (Figure 3B). We aimed to determine whether these distantly related ssRNAPs also catalyze the DNA-terminus-dependent transcription and produce the full-length dsRNA. With DNA templates harboring the same GFP coding sequence, we compared the production of the full-length dsRNA by these ssRNAPs to that by T7 and SP6 RNAPs (39). Interestingly, among the five ssRNAPs investigated, KP34 and VSW-3 RNAPs do not produce detectable full-length dsRNA (Figure 3C and D), indicating that the ssRNAPs from phiKMV-like viruses might not initiate transcription from DNA termini. Thus, DNA-terminus-initiated, promoter-independent transcription is not a common feature for bacteriophage ssRNAPs.

### T7 RNAP mutants with low full-length dsRNA production

**Figure 4.**
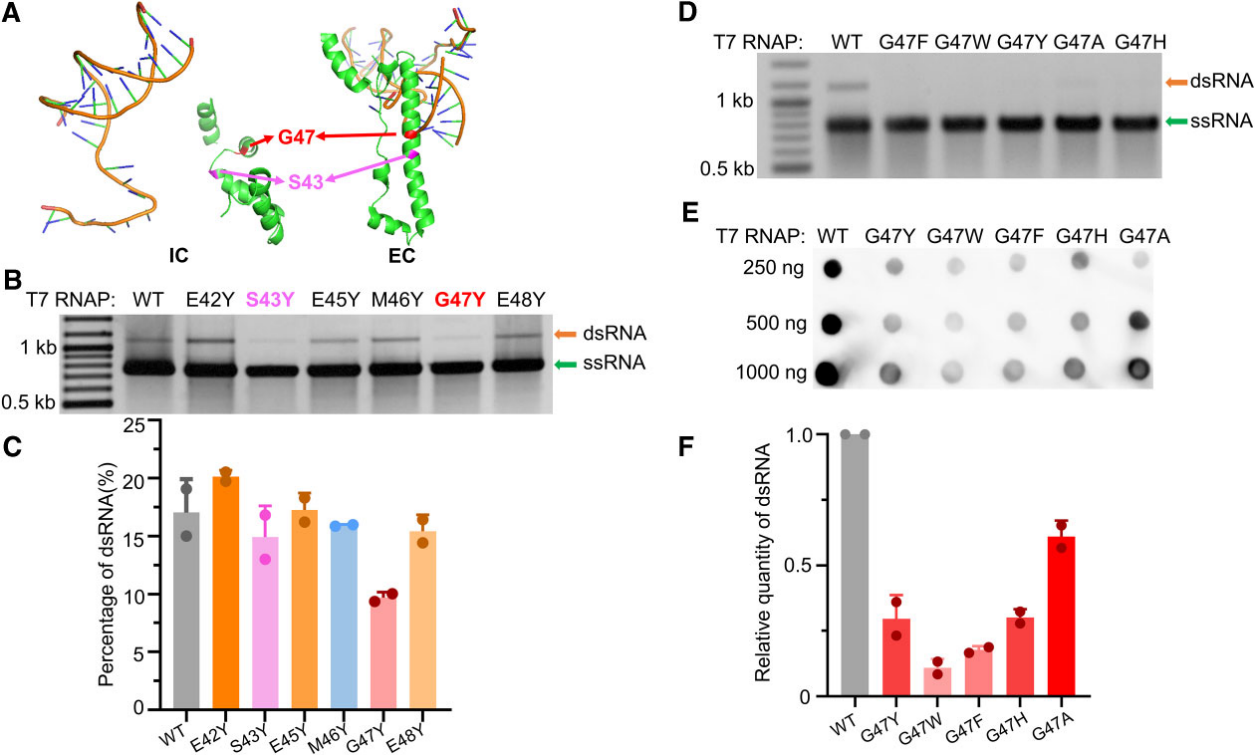
C-helix mutations in T7 RNAP affect the production of full-length dsRNA. (**A**) Structures of the C-helix in the T7 RNAP initiation complex (IC, PDB: 2PI4) and elongation complex (EC, PDB: 1MSW). Residue G47 and S43 are indicated by arrows. (**B**) Agarose gel electrophoresis analysis of GFP transcripts synthesized by WT T7 RNAP and its mutants E42Y, S43Y, E45Y, M46Y, G47Y and E48Y in IVT. (**C**) Quantification of the results shown in panel (B). The gray values of the gel bands corresponding to the runoff ssRNA and the full-length dsRNA were measured, and the percentage of the full-length dsRNA was calculated as the ratio between the full-length dsRNA and the sum of the runoff ssRNA and the full-length dsRNA. (**D**) Agarose gel electrophoresis analysis of GFP transcripts synthesized by WT T7 RNAP and its mutants G47F, G47W, G47Y, G47H and G47A in IVT. (**E**) Dot blot analysis of dsRNA contents from samples in panel (D). (**F**) Quantification of the full-length dsRNA shown in panel (E). dsRNA content was normalized to that obtained using WT T7 RNAP. Gel bands corresponding to ssRNA and dsRNA are indicated by arrows, respectively, in all gels.

Previous efforts (2,17–32) have been made to eliminate the dsRNA by-products in IVT, which is the major source of immunogenicity for mRNA therapeutics. However, these works have barely focused on the dsRNA generated by promoter-independent antisense transcription. The fact that KP34 and VSW-3 RNAPs do not initiate transcription from DNA termini encouraged us to engineer T7 RNAP to reduce its DNA-terminus-dependent transcription. Previous studies (31,32,35,36) suggested that the C-helix (residues 28–71) of T7 RNAP is important for the transition from initiation to elongation, and contacts the DNA template in the elongation complex. The C-helix consists of two helices that are hinged to allow bending to create one longer helix during the transition from transcription initiation to elongation, with drastic conformational changes occurring at the two hinge residues, Ser43 and Gly47 (Figure 4A). Dousis *et al.* reported that alanine substitution at position 47 of T7 RNAP is the most advantageous for the formation of the C-helix, thus reducing the production of self-extended dsRNA to the highest extent (32). Our previous work applying directed evolution revealed that a tyrosine substitution at position 43 of T7 RNAP causes the most significant reduction of the terminal self-extended dsRNA (31). Hence, we performed a ‘tyrosine screen’ for residues 42–48 by substituting each of the residues with tyrosine and examined the IVT products by these T7 RNAP mutants on GFP templates (Figure 4B). Interestingly, tyrosine substitutions at these positions showed various effects on the production of full-length dsRNA: E42Y and E45Y mutations increased the production of full-length dsRNA and S43Y slightly reduced the production of full-length dsRNA, while G47Y significantly reduced the production of full-length dsRNA (Figure 4C). To further confirm whether aromatic residues at position 47 cause the observed effect, we mutated G47 to Tyr, Trp, Phe, His and Ala and analyzed their effects on the production of full-length dsRNA (Figure 4D and E). All these mutations reduced the production of full-length dsRNA by T7 RNAP, with aromatic residues showing the strongest effects (Figure 4F). Among them, the G47W mutant, with the largest residue at position 47, produced the least full-length dsRNA. The amount of full-length dsRNA in the GFP transcripts produced by the G47W mutant was reduced 9-fold compared with that produced by WT T7 RNAP (Figure 4F).

### T7 RNAP mutants with high full-length dsRNA production

**Figure 5.**
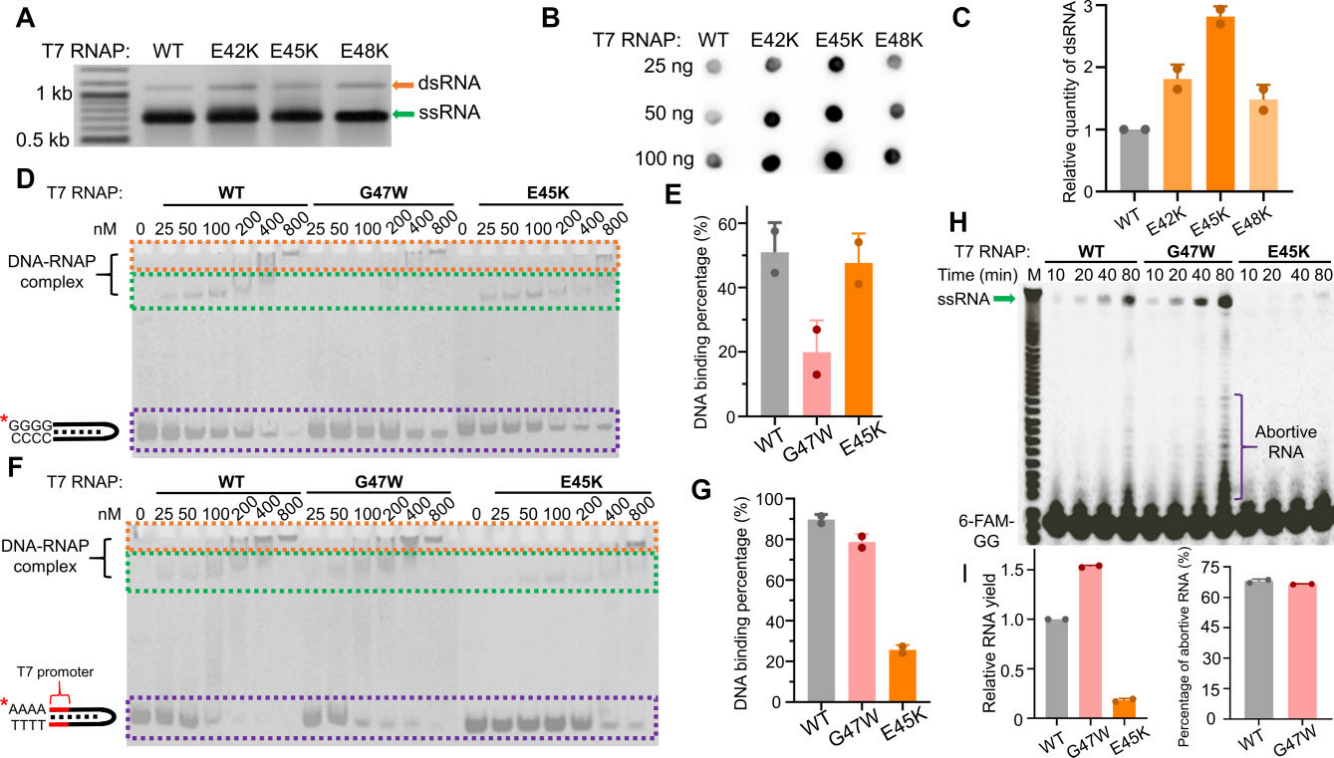
C-helix mutations affect the DNA binding by T7 RNAP. (**A**) Agarose gel electrophoresis analysis of GFP transcripts synthesized by WT T7 RNAP and its mutants E42K, E45K and E48K. (**B**) Dot blot analysis of dsRNA contents from samples in panel (A). (**C**) Quantification of the full-length dsRNA shown in panel (B). dsRNA content was normalized to that obtained using WT T7 RNAP. (**D**) Binding of WT T7 RNAP or its G47W or E45K mutant to a 5′-6-FAM (indicated by the asterisk) fluorescently tagged DNA hairpin without T7 promoter. The 5′ terminal sequence of the DNA hairpin was GGGG to enhance the DNA-terminus binding of T7 RNAP. The incubated complexes were analyzed by 10% native PAGE, and the bands corresponding to the unbound DNA, the bound DNA, and the large complex are enclosed by bottom, middle and top dashed box, respectively. (**E**) DNA binding percentages of WT T7 RNAP or its G47W orE45K mutant at 200 nM enzyme concentration in panel (D). The gray values of the gel bands corresponding to the unbound DNA were measured, and the percentage of bound DNA was calculated as the reduction of unbound DNA compared to that of the no protein group ‘0’. (**F**) Binding of WT T7 RNAP or its G47W or E45K mutant to a 5′-6-FAM (indicated by the asterisk) fluorescently tagged DNA hairpin with a T7 promoter. The 5′ terminal sequence of the DNA hairpin was AAAA to minimize the DNA-terminus binding of T7 RNAP. The incubated complexes were analyzed by 10% native PAGE, and the bands corresponding to the unbound DNA, the bound DNA, and the large complex are enclosed by bottom, middle and top dashed box, respectively. (**G**) DNA binding percentages of WT T7 RNAP or its G47W orE45K mutant at 200 nM enzyme concentration in panel (F). The percentage of bound DNA was calculated as in panel (E). (**H**) 20% denaturing PAGE analysis of the IVT transcripts from WT T7 RNAP or its G47W or E45K mutant at the indicated time points. IVT reactions were initiated by the fluorescently labeled dinucleotide 6-FAM-GG, and results were visualized by fluorescence imaging. The runoff RNA, abortive RNA and unincorporated 6-FAM-GG are marked. (**I**) Quantification of the runoff RNA shown in panel (H). The gray values of the gel bands corresponding to the runoff RNA and the abortive RNA were measured. Runoff RNA yield was normalized to that obtained using WT T7 RNAP in the left panel. The percentage of abortive RNA was calculated as the ratio between abortive RNA and the sum of the abortive RNA and the runoff RNA. The percentage of abortive RNA was normalized to the percentage obtained using WT T7 RNAP in the right panel. The abortive RNA obtained using the E45K mutant was undetectable. Gel bands corresponding to ssRNA and dsRNA are indicated by arrows, respectively, in all gels.

In contrast to G47Y, mutations E42Y and E45Y increased the production of full-length dsRNA (Figure 4C). We also replaced the three negatively charged glutamic acids E42, E45 and E48 in the C-helix with positively charged lysine residues. Both gel electrophoresis (Figure 5A) and dot blot analyses (Figure 5B and C) showed significant increases in the production of full-length dsRNA in the IVT of GFP RNA by E42K, E45K and E48K mutants compared to that by the WT T7 RNAP. The most significant increase was observed for the E45K mutant (Figure 5C). To elucidate the mechanism by which G47W and E45K mutations affect the DNA-terminus-dependent transcription by T7 RNAP, we designed a 5′-6-FAM fluorescently labeled 43-bp DNA hairpin with four consecutive Gs at the 5′-terminus lacking T7 promoter (Figure 5D) and investigated the binding of such DNA template by the WT T7 RNAP and its G47W and E45K mutants. WT, G47W or E45K T7 RNAP was incubated with the promoter-less DNA, and the binding of the enzymes to the DNA was analyzed by 10% native PAGE. The fluorescence gel image was obtained using the 492- and 517-nm filters for excitation and emission, respectively, and the image was converted into black/white (Figure 5D). At low enzyme concentrations (≤ 200 nM), the electrophoresis mobility shift assay (EMSA) results showed weak but specific slowly moving gel bands for WT T7 RNAP and the E45K mutant (Figure 5D, middle dashed box), indicating promoter-independent binding to the DNA terminus. Such binding is much weaker for the G47W mutant compared to that of the WT T7 RNAP (Figure 5D, middle dashed box), consistent with its low full-length dsRNA production in IVT. At 200 nM enzyme concentration, the DNA-terminus binding of WT T7 RNAP and its C-helix mutants was quantified and compared (Figure 5E).

We also investigated the effect of G47W or E45K mutation on the normal functions of T7 RNAP. First, the binding of WT and mutant RNAPs to a 5′-6-FAM fluorescently labeled 43-bp DNA hairpin containing a T7 promoter was evaluated. The 5′-terminus of such DNA hairpin was designed as four consecutive As to minimize the terminus binding (Figure 5F). Inconsistent with the binding to DNA terminus, the binding to the T7 promoter was slightly weakened by the G47W mutation (Figure 5F, middle dashed box). At 200 nM enzyme concentration, the promoter binding of WT T7 RNAP and its C-helix mutants was quantified and compared (Figure 5G). While unexpectedly the E45K mutation decreased the binding of T7 RNAP to its promoter (Figure 5F and G). Since the promoter binding and nonpromoter binding are in competition, the weakened promoter binding of E45K might be responsible for its increased dsRNA synthesis. These results indicate different modes of promoter or DNA terminus binding by T7 RNAP.

We noticed that for both DNA substrates tested in the EMSA, at high enzyme concentrations (above 200 nM), the gel bands indicating DNA binding were retarded close to the loading wells (Figure 5D and F, top dashed box), indicating formation of large complex. Intriguingly, this binding was independent of either the DNA promoter or terminus, suggesting that another binding mode to DNA distinct from the promoter and terminus binding modes exists. The E45K mutation attenuated the formation of large complex at high enzyme concentrations (Figure 5D and F, top dashed box). The mechanism underlying this unknown DNA binding by T7 RNAP is to be investigated.

Moreover, as conformational changes of the C-helix occur during the transition from transcription initiation to elongation (35), we also investigated the influence of G47W or E45K mutation on this step. T7 RNAP is known to initiate transcription efficiently with dinucleotides matching the initial RNA sequences (41–43), so we designed a 53-bp template with a T7 promoter, followed by three consecutive Gs, and added the fluorescently labeled dinucleotide 6-FAM-GG into the reactions to initiate the IVT by WT T7 RNAP or its G47W or E45K mutant. At 10, 20, 40 and 80 min after initiation of the reactions, 0.5 μl of every sample was taken and analyzed by 20% denaturing PAGE. Then, fluorescence gel image of RNA transcripts was obtained using the 492- and 517-nm filters for excitation and emission, respectively, and the image was converted into black/white (Figure 5H). We quantified the yield of runoff ssRNA and the abortive RNA products. The result demonstrated that the G47W mutant initiates transcription more efficiently with the dinucleotide GG compared to WT T7 RNAP to produce more runoff and abortive RNA (Figure 5I). However, the ratio between the abortive and runoff products was not significantly changed by the G47W mutation (Figure 5H and I), indicating that the transition from transcription initiation to elongation was not affected. In contrast, the E45K mutation severely reduced the initiation and the yield in IVT (Figure 5H and I), consistent with its effect on promoter binding.

### T7 RNAP-G47W reduces the dsRNA by-products for mRNA production

**Figure 6.**
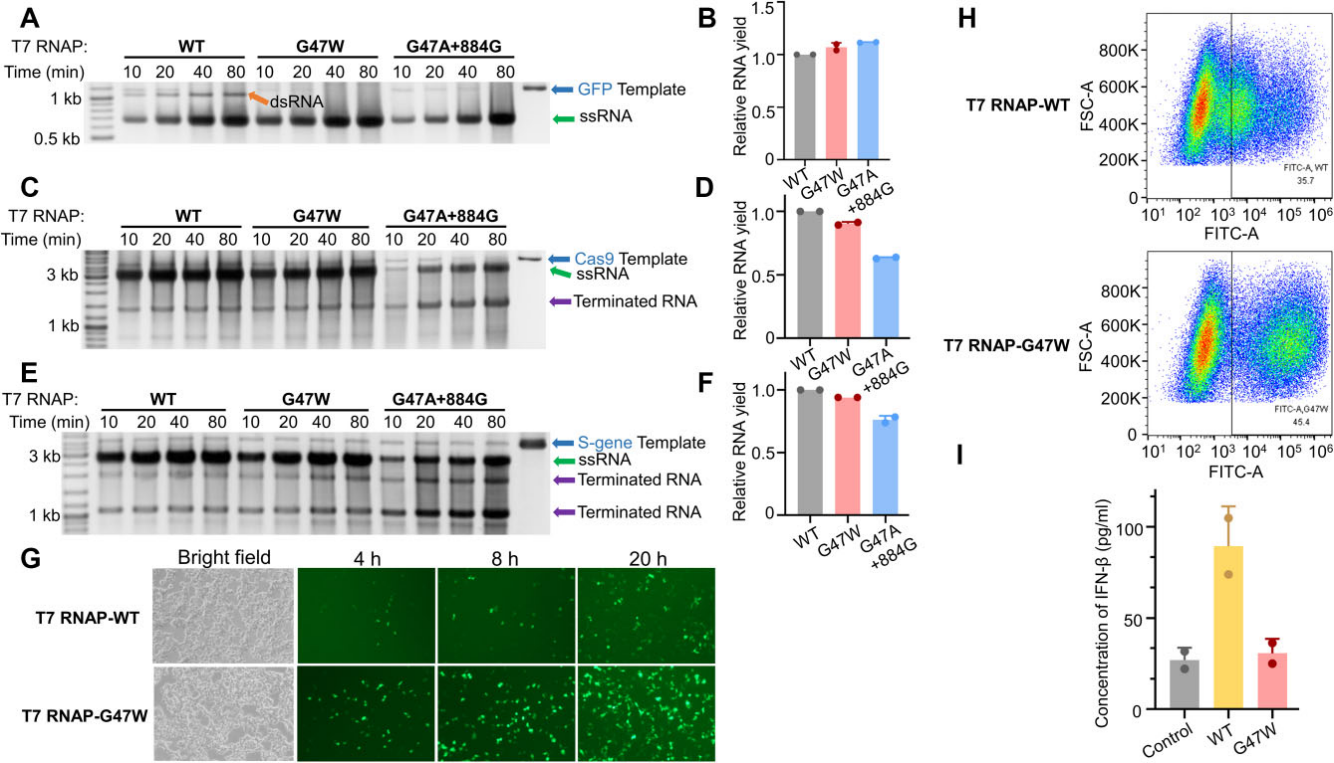
T7 RNAP-G47W is advantageous for mRNA synthesis. (**A**) 1.5% agarose gel electrophoresis analysis of the IVT production of GFP RNA by T7 RNAP (WT) or its G47W or G47A + 884G mutant. (**B**) Quantification of the runoff ssRNA shown in panel (A). The gray values of the gel bands corresponding to the runoff ssRNA were measured. The runoff ssRNA yield was normalized to that obtained using WT T7 RNAP. (**C**) 1.5% agarose gel electrophoresis analysis of the IVT production of Cas9 RNA by T7 RNAP (WT) or its G47W or G47A + 884G mutant. (**D**) Quantification of the runoff ssRNA shown in panel (C). (**E**) 1.5% agarose gel electrophoresis analysis of the IVT production of S-gene RNA by T7 RNAP (WT) or its G47W or G47A + 884G mutant. (**F**) Quantification of the runoff ssRNA shown in panel (E). DNase I treatment was omitted for all reactions shown in panels (A), (C) and (E), and the DNA template is shown as a loading control. DNA templates, ssRNA, dsRNA and terminated RNA are indicated by arrows, respectively, in panels (A), (C), and (E). (**G**) Expression levels of GFP mRNA produced by T7 RNAP-WT or T7 RNAP-G47W in HEK293T cells recorded by fluorescence microscopy at 4, 16 and 20 h after transfection. (**H**) Fluorescence intensity of cells transfected with GFP mRNA synthesized by T7 RNAP-WT or T7 RNAP-G47W at 20 h after transfection, as determined by flow cytometry. (**I**) Concentrations of IFN-β in cells transfected with GFP mRNA synthesized by T7 RNAP-WT or T7 RNAP-G47W at 20 h after transfection. The cells treated with Lipofectamine 2000 alone served as the control.

To evaluate the advantages of T7 RNAP-G47W in mRNA production, we compared WT T7 RNAP and T7 RNAP-G47W in the IVT production of GFP, Cas9 and S-gene RNA. Another mutant of T7 RNAP, G47A + 884G, which was recently reported to significantly reduce the self-templated dsRNA (32), was also included in the comparison. For both the G47W and G47A + 884G mutants, the gel bands corresponding to the full-length dsRNA in the GFP IVT products were not observable (Figure 6A), indicating that DNA-terminus-initiated transcription by the G47A + 884G mutant is also reduced compared to that by the WT T7 RNAP. The IVT yield of GFP RNA, with a relatively short length (873 nt), was similar for WT T7 RNAP and both mutants (Figure 6A and B). However, when synthesizing large RNA molecules like Cas9 (4314 nt) and S-gene (3975 nt), the IVT yield of the G47W mutant was slightly lower than that of WT T7 RNAP, while the IVT yield of the G47A + 884G mutant was obviously reduced (Figure 6C–F). In addition, the G47A + 884G mutant produced more terminated products compared to the WT and G47W T7 RNAPs (Figure 6C and E, indicated by arrows).

To compare the expression efficiency of mRNA produced by WT T7 RNAP and the G47W mutant, we transfected HEK293T cells with GFP mRNA synthesized by either enzyme, and their expression levels were recorded by fluorescence microscopy at 4, 16 and 20 h after transfection (Figure 6G). After 20 h, we quantified their fluorescence intensity by flow cytometry (Figure 6H). As expected, mRNA transcribed by the G47W mutant showed higher expression level than that transcribed by WT T7 RNAP. We also determined the concentration of IFN-β in HEK293T cells at 20 h after transfection by ELISA and found that almost no IFN-β response was induced in the cells transfected with GFP mRNA produced by the G47W mutant compared to the negative control (Figure 6I). However, the mRNA produced by WT T7 RNAP elicited a strong IFN-β response. These results are consistent with the low production of full-length dsRNA by the T7 RNAP-G47W mutant. It should be noted that uncharacterized factors other than the dsRNA contents may also influence the results of these assays.

## Discussion

It is well known that mRNA transcribed by T7 RNAP can stimulate the mammalian innate immune system and that it is necessary to reduce the immunostimulatory effect of the dsRNA to fit therapeutic applications. Previous studies (9–11,13) focused on the dsRNA generated by self-template extension. Recently, Mu *et al.* reported that WT T7 RNAP can initiate transcription from the end of the DNA in a promoter-independent manner to generate full-length dsRNA and that this transcription could be suppressed by low concentrations of Mg^2+^ (15). Yet, the mechanisms underlying such nonconventional transcription remain elusive.

In the present study, we further analyzed the promoter-independent transcription by T7 RNAP and revealed more details about the process. We demonstrated that the production of antisense RNA is mostly initiated from the penultimate position of the DNA terminus and that the presence of guanosine or cytidine in the 2-nt terminal region of the DNA template strengthens the promoter-independent transcription by T7 RNAP significantly. Therefore, adding poly(A) to the end of DNA templates is an effective way to reduce the amounts of full-length dsRNA in mRNA production.

Moreover, we tested the full-length dsRNA production by various ssRNAPs and found that DNA-terminus-dependent transcription is not common for bacteriophage ssRNAPs. Although T7, SP6 (39) and Syn5 (37,38) RNAPs as representative ssRNAPs all produce significant amounts of full-length dsRNA in IVT, the products of KP34 (27) and VSW-3 (28,29) RNAPs from phiKMV-like viruses (40) contain nondetectable dsRNA generated by promoter-independent transcription.

Previous studies proved that mutations S43Y and G47A in T7 RNAP were able to attenuate the self-template extension (31,32). We replaced residues 42–48 of T7 RNAP with various amino acids, and the results showed that substitutions of G47 with large amino acids such as aromatic amino acids most significantly reduce the dsRNA generated from promoter-independent transcription. In contrast, substitutions of negatively charged E42, E45 or E48 with lysine enhanced the promoter-independent transcription. Moreover, we demonstrated that the G47W and E45K mutants affected the DNA binding of T7 RNAP. As the direct interaction between DNA template and the C-helix in the elongation complex occurs several amino acids away from G47 and E45 (36), the impact of their substitutions on DNA interaction is likely an indirect effect, presumably on the rigidity of the C-helix. In summary, our results demonstrate the importance of residues 42–48 of T7 RNAP for the interaction with DNA; these residues may serve as potential targets for further engineering.

## Supplementary Material

gkae593_Supplemental_File

## Data Availability

The data underlying this article are available in the article and in its online supplementary material.
