# Supplementary material for: DNA-terminus-dependent transcription by T7 RNA polymerase and its C-helix mutants
Source: Nucleic Acids Res. 2024 Jul 9;52(14):8443–53. doi: 10.1093/nar/gkae593 (PMC11317132; doi:10.1093/nar/gkae593)
Supplement: gkae593_Supplemental_File [file gkae593_supplemental_file.pdf]

Supplementary Materials

Table S1. Sequences of the DNA templates and primers used in this study.

DNA	Sequence (5′-3′)
Gfp	ATGGCTAGCAAAGGAGAAGAACTCTTCACTGGAGTTGTCCCAATTC<br>TTGTTGAATTAGATGGTGATGTTAACGGCCACAAGTTCTCTGTCAGT<br>GGAGAGGGTGAAGGTGATGCAACATACGGAAAACTTACCCTGAAGT<br>TCATCTGCACTACTGGCAAACTGCCTGTTCCATGGCCAACACTAGTC<br>ACTACTCTGTGCTATGGTGTTCAATGCTTTTCAAGATACCCGGATCAT<br>ATGAAACGGCATGACTTTTTCAAGAGTGCCATGCCCGAAGGTTATGT<br>ACAGGAAAGGACCATCTTCTTCAAAGATGACGGCAACTACAAGACA<br>CGTGCTGAAGTCAAGTTTGAAGGTGATACCCTTGTTAATAGAATCGA<br>GTTAAAAGGTATTGACTTCAAGGAAGATGGCAACATTCTGGGACAC<br>AAATTGGAATACAACTATAACTCACACAATGTATACATCATGGCAGAC<br>AAACAAAAGAATGGAATCAAAGTGAACTTCAAGACCCGCCACAAC<br>ATTGAAGATGGAAGCGTTCAACTAGCAGACCATTATCAACAAAATAC<br>TCCAATTGGCGATGGCCCTGTCCTTTTACCAGACAACCATTACCTGTC<br>CACACAATCTGCCCTTTCGAAAGATCCCAACGAAAAGAGAGACCAC<br>ATGGTCCTTCTTGAGTTTGTAACAGCTGCTGGGATTACACATGGCAT<br>GGATGAACTGTACAACTGA
SOX7	ATGAAAAGGCCGGCGGCCACGAAAAAGGCCGGCCAGGCAAAAAAG<br>AAAAAGGGTTCTGGAGCTTCGCTGCTGGGAGCCTACCCTTGGCCCG<br>AGGGTCTCGAGTGCCCGGCCCTGGACGCCGAGCTGTCGGATGGACA<br>ATCGCCGCCGGCCGTCCCCCGGCCCCCGGGGGACAAGGGCTCCGAG<br>AGCCGTATCCGGCGGCCCATGAACGCCTTCATGGTTTGGGCCAAGG<br>ACGAGAGGAAACGGCTGGCAGTGCAGAACCCGGACCTGCACAACG<br>CCGAGCTCAGCAAGATGCTGGGAAAGTCGTGGAAGGCGCTGACGCT<br>GTCCCAGAAGAGGCCGTACGTGGACGAGGCGGAGCGGCTGCGCCT<br>GCAGCACATGCAGGACTACCCCAACTACAAGTACCGGCCGCGCAGG<br>AAGAAGCAGGCCAAGCGGCTGTGCAAGCGCGTGGACCCGGGCTTC<br>CTTCTGAGCTCCCTCTCCCGGGACCAGAACGCCCTGCCGGAGAAGA<br>GAAGCGGCAGCCGGGGGGCGCTGGGGGAGAAGGAGGACAGGGGT<br>GAGTACTCCCCCGGCACTGCCCTGCCCAGCCTCCGGGGCTGCTACC<br>ACGAGGGGCCGGCTGGTGGTGGCGGCGGCGGCACCCCGAGCAGTG<br>TGGACACGTACCCGTACGGGCTGCCCACACCTCCTGAAATGTCTCCC<br>CTGGACGTGCTGGAGCCGGAGCAGACCTTCTTCTCCTCCCCCTGCC<br>AGGAGGAGCATGGCCATCCCCGCCGCATCCCCCACCTGCCAGGGCA<br>CCCGTACTCACCGGAGTACGCCCCAAGCCCTCTCCACTGTAGCCACC<br>CCCTGGGCTCCCTGGCCCTTGGCCAGTCCCCCGGCGTCTCCATGATG<br>TCCCCTGTACCCGGCTGTCCCCCATCTCCTGCCTATTACTCCCCGGCC<br>ACCTACCACCCACTCCACTCCAACCTCCAAGCCCACCTGGGCCAGC<br>TTTCCCCGCCTCCTGAGCACCCTGGCTTCGACGCCCTGGATCAACTG<br>AGCCAGGTGGAACTCCTGGGGGACATGGATCGCAATGAATTCGACC<br>AGTATTTGAACACTCCTGGCCACCCAGACTCCGCCACAGGGGCCAT<br>GGCCCTCAGTGGGCATGTTCCGGTCTCCCAGGTGACACCAACGGGT

	CCCACAGAGACCAGCCTCATCTCCGTCCTGGCTGATGCCACGGCCA CGTACTACAACAGCTACAGTGTGTCATGA
S-gene	ATGTTTGTTTTTCTTGTTTTATTGCCACTAGTCTCTAGTCAGTGTGTTA ATCTTACAACCAGAACTCAATTACCCCCTGCATACACTAATTCTTTCA CACGTGGTGTTTATTACCCTGACAAAGTTTTCAGATCCTCAGTTTTAC ATTCAACTCAGGACTTGTTCTTACCTTTCTTTTCCAATGTTACTTGGT TCCATGCTATACATGTCTCTGGGACCAATGGTACTAAGAGGTTTGATA ACCCTGTCCTACCATTTAATGATGGTGTTTATTTTGCTTCCACTGAGA AGTCTAACATAATAAGAGGCTGGATTTTTGGTACTACTTTAGATTCGA AGACCCAGTCCCTACTTATTGTTAATAACGCTACTAATGTTGTTATTAA AGTCTGTGAATTTCAATTTTGTAATGATCCATTTTTGGGTGTTTATTAC CACAAAAACAACAAAAGTTGGATGGAAAGTGAGTTCAGAGTTTATT CTAGTGCGAATAATTGCACTTTTGAATATGTCTCTCAGCCTTTTCTTAT GGACCTTGAAGGAAAACAGGGTAATTTCAAAAATCTTAGGGAATTT GTGTTTAAGAATATTGATGGTTATTTTAAAATATATTCTAAGCACACGC CTATTAATTTAGTGCGTGATCTCCCTCAGGGTTTTTCGGCTTTAGAAC CATTGGTAGATTTGCCAATAGGTATTAACATCACTAGGTTTCAAACTT TACTTGCTTTACATAGAAGTTATTTGACTCCTGGTGATTCTTCTTCAG GTTGGACAGCTGGTGCTGCAGCTTATTATGTGGGTTATCTTCAACCTA GGACTTTTCTATTAAAATATAATGAAAATGGAACCATTACAGATGCTG TAGACTGTGCACTTGACCCTCTCTCAGAAACAAAGTGTACGTTGAA ATCCTTCACTGTAGAAAAAGGAATCTATCAAACTTCTAACTTTAGAG TCCAACCAACAGAATCTATTGTTAGATTTCCTAATATTACAAACTTGT GCCCTTTTGGTGAAGTTTTTAACGCCACCAGATTTGCATCTGTTTATG CTTGGAACAGGAAGAGAATCAGCAACTGTGTTGCTGATTATTCTGTC CTATATAATTCCGCATCATTTTCCACTTTTAAGTGTTATGGAGTGTCTC CTACTAAATTAAATGATCTCTGCTTTACTAATGTCTATGCAGATTCATT TGTAATTAGAGGTGATGAAGTCAGACAAATCGCTCCAGGGCAAACT GGAAAGATTGCTGATTATAATTATAAATTACCAGATGATTTTACAGGC TGCGTTATAGCTTGGAATTCTAACAATCTTGATTCTAAGGTTGGTGGT AATTATAATTACCTGTATAGATTGTTTAGGAAGTCTAATCTCAAACCTT TTGAGAGAGATATTTCAACTGAAATCTATCAGGCCGGTAGCACACCT TGTAATGGTGTTGAAGGTTTTAATTGTTACTTTCCTTTACAATCATATG GTTTCCAACCCACTAATGGTGTTGGTTACCAACCATACAGAGTAGTA GTACTTTCTTTTGAACTTCTACATGCACCAGCAACTGTTTGTGGACCT AAAAAGTCTACTAATTTGGTTAAAAACAAATGTGTCAATTTCAACTT CAATGGTTTAACAGGCACAGGTGTTCTTACTGAGTCTAACAAAAAGT TTCTGCCTTTCCAACAATTTGGCAGAGACATTGCTGACACTACTGAT GCTGTCCGTGATCCACAGACACTTGAGATTCTTGACATTACACCATG TTCTTTTGGTGGTGTCAGTGTTATAACACCAGGAACAAATACTTCTA ACCAGGTTGCTGTTCTTTATCAGGATGTTAACTGCACAGAAGTCCCT GTTGCTATTCATGCAGATCAACTTACTCCTACTTGGCGTGTTTATTCTA CAGGTTCTAATGTTTTTCAAACACGTGCAGGCTGTTTAATAGGGGCT GAACATGTCAACAACTCATATGAGTGTGACATACCCATTGGTGCAGG

	TATATGCGCTAGTTATCAGACTCAGACTAATTCTCCTCGGCGGGCACG<br>TAGTGTAGCTAGTCAATCCATCATTGCCTACACTATGTCACTTGGTGC<br>AGAAAATTCAGTTGCTTACTCTAATAACTCTATTGCCATACCCACAAA<br>TTTTACTATTAGTGTTACCACAGAAATTCTACCAGTGTCTATGACCAA<br>GACATCAGTAGATTGTACAATGTACATTTGTGGTGATTCAACTGAATG<br>CAGCAATCTTTTGTTGCAATATGGCAGTTTTTGTACACAATTAAACCG<br>TGCTTTAACTGGAATAGCTGTTGAACAAGACAAAAACACCCAAGAA<br>GTTTTTGCACAAGTCAAACAAATTTACAAAACACCACCAATTAAAG<br>ATTTTGGTGGTTTTAATTTTTCACAAATATTACCAGATCCATCAAAAC<br>CAAGCAAGAGGTCATTTATTGAAGATCTACTTTTCAACAAAGTGACA<br>CTTGCAGATGCTGGCTTCATCAAACAATATGGTGATTGCCTTGGTGAT<br>ATTGCTGCTAGAGACCTCATTTGTGCACAAAAGTTTAACGGCCTTAC<br>TGTTTTGCCACCTTTGCTCACAGATGAAATGATTGCTCAATACACTTC<br>TGCACTGTTAGCGGGTACAATCACTTCTGGTTGGACCTTTGGTGCAG<br>GTGCTGCATTACAAATACCATTTGCTATGCAAATGGCTTATAGGTTTA<br>ATGGTATTGGAGTTACACAGAATGTTCTCTATGAGAACCAAAAATTG<br>ATTGCCAACCAATTTAATAGTGCTATTGGCAAAATTCAAGACTCACTT<br>TCTTCCACAGCAAGTGCACTTGGAAAACTTCAAGATGTGGTCAACC<br>AAAATGCACAAGCTTTAAACACGCTTGTTAAACAACTTAGCTCCAAT<br>TTTGGTGCAATTTCAAGTGTTTTAAATGATATCCTTTCACGTCTTGAC<br>AAAGTTGAGGCTGAAGTGCAAATTGATAGGTTGATCACAGGCAGAC<br>TTCAAAGTTTGCAGACATATGTGACTCAACAATTAATTAGAGCTGCA<br>GAAATCAGAGCTTCTGCTAATCTTGCTGCTACTAAAATGTCAGAGTG<br>TGTACTTGGACAATCAAAAAGAGTTGATTTTTGTGGAAAGGGCTATC<br>ATCTTATGTCCTTCCCTCAGTCAGCACCTCATGGTGTAGTCTTCTTGC<br>ATGTGACTTATGTCCCTGCACAAGAAAAGAACTTCACAACTGCTCCT<br>GCCATTTGTCATGATGGAAAAGCACACTTTCCTCGTGAAGGTGTCTT<br>TGTTTCAAATGGCACACACTGGTTTGTAACACAAAGGAATTTTTATG<br>AACCACAAATCATTACTACAGACAACACATTTGTGTCTGGTAACTGT<br>GATGTTGTAATAGGAATTGTCAACAACACAGTTTATGATCCTTTGCAA<br>CCTGAATTAGACTCATTCAAGGAGGAGTTAGATAAATATTTTAAGAAT<br>CATACATCACCAGATGTTGATTTAGGTGACATCTCTGGCATTAATGCT<br>TCAGTTGTAAACATTCAAAAAGAAATTGACCGCCTCAATGAGGTTGC<br>CAAGAATTTAAATGAATCTCTCATCGATCTCCAAGAACTTGGAAAGT<br>ATGAGCAGTATATAAAATGGCCATGGTACATTTGGCTAGGTTTTATAG<br>CTGGCTTGATTGCCATAGTAATGGTGACAATTATGCTTTGCTGTATGA<br>CCAGTTGCTGTAGTTGTCTCAAGGGCTGTTGTTCTTGTGGATCCTGC<br>TGCAAATTTGATGAAGACGACTCTGAGCCAGTGCTCAAAGGAGTCA<br>AATTACATTACACATAA
Cas9	ATGAAAAGGCCGGCGGCCACGAAAAAGGCCGGCCAGGCAAAAAAG<br>AAAAAGGGTTCTGGAGATAAAAAGTATTCTATTGGTTTAGACATCGG<br>CACTAATTCCGTTGGATGGGCTGTCATAACCGATGAATACAAAGTAC<br>CTTCAAAGAAATTTAAGGTGTTGGGGAACACAGACCGTCATTCGATT<br>AAAAAGAATCTTATCGGTGCCCTCCTATTCGATAGTGGCGAAACGGC

	AGAGGCGACTCGCCTGAAACGAACCGCTCGGAGAAGGTATACACGT<br>CGCAAGAACCGAATATGTTACTTACAAGAAATTTTTAGCAATGAGAT<br>GGCCAAAGTTGACGATTCTTTCTTTCACCGTTTGGAAGAGTCCTTCC<br>TTGTCGAAGAGGACAAGAAACATGAACGGCACCCCATCTTTGGAAA<br>CATAGTAGATGAGGTGGCATATCATGAAAAGTACCCAACGATTTATCA<br>CCTCAGAAAAAAGCTAGTTGACTCAACTGATAAAGCGGACCTGAGG<br>TTAATCTACTTGGCTCTTGCCCATATGATAAAGTTCCGTGGGCACTTT<br>CTCATTGAGGGTGATCTAAATCCGGACAACTCGGATGTCGACAAACT<br>GTTCATCCAGTTAGTACAAACCTATAATCAGTTGTTTGAAGAGAACC<br>CTATAAATGCAAGTGGCGTGGATGCGAAGGCTATTCTTAGCGCCCGC<br>CTCTCTAAATCCCGACGGCTAGAAAACCTGATCGCACAATTACCCGG<br>AGAGAAGAAAAATGGGTTGTTCGGTAACCTTATAGCGCTCTCACTAG<br>GCCTGACACCAAATTTTAAGTCGAACTTCGACTTAGCTGAAGATGCC<br>AAATTGCAGCTTAGTAAGGACACGTACGATGACGATCTCGACAATCT<br>ACTGGCACAAATTGGAGATCAGTATGCGGACTTATTTTTGGCTGCCA<br>AAAACCTTAGCGATGCAATCCTCCTATCTGACATACTGAGAGTTAATA<br>CTGAGATTACCAAGGCGCCGTTATCCGCTTCAATGATCAAAAGGTAC<br>GATGAACATCACCAAGACTTGACACTTCTCAAGGCCCTAGTCCGTCA<br>GCAACTGCCTGAGAAATATAAGGAAATATTCTTTGATCAGTCGAAAA<br>ACGGGTACGCAGGTTATATTGACGGCGGAGCGAGTCAAGAGGAATT<br>CTACAAGTTTATCAAACCCATATTAGAGAAGATGGATGGGACGGAAG<br>AGTTGCTTGTAAAACTCAATCGCGAAGATCTACTGCGAAAGCAGCG<br>GACTTTCGACAACGGTAGCATTCCACATCAAATCCACTTAGGCGAAT<br>TGCATGCTATACTTAGAAGGCAGGAGGATTTTTATCCGTTCCTCAAAG<br>ACAATCGTGAAAAGATTGAGAAAATCCTAACCTTTCGCATACCTTAC<br>TATGTGGGACCCCTGGCCCGAGGGAACTCTCGGTTCGCATGGATGAC<br>AAGAAAGTCCGAAGAAACGATTACTCCCTGGAATTTTGAGGAAGTT<br>GTCGATAAAGGTGCGTCAGCTCAATCGTTCATCGAGAGGATGACCGC<br>CTTTGACAAGAATTTACCGAACGAAAAAGTATTGCCTAAGCACAGTT<br>TACTTTACGAGTATTTCACAGTGTACAATGAACTCACGAAAGTTAAG<br>TATGTCACTGAGGGCATGCGTAAACCCGCCTTTCTAAGCGGAGAACA<br>GAAGAAAGCAATAGTAGATCTGTTATTCAAGACCAACCGCAAAGTG<br>ACAGTTAAGCAATTGAAAGAGGACTACTTTAAGAAAATTGAATGCTT<br>CGATTCTGTCGAGATCTCCGGGGTAGAAGATCGATTTAATGCGTCAC<br>TTGGTACGTATCATGACCTCCTAAAGATAATTAAAGATAAGGACTTCC<br>TGGATAACGAAGAGAATGAAGATATCTTAGAAGATATAGTGTTGACT<br>CTTACCCTCTTTGAAGATCGGGAAATGATTGAGGAAAGACTAAAAA<br>CATACGCTCACCTGTTCGACGATAAGGTTATGAAACAGTTAAAGAGG<br>CGTCGCTATACGGGCTGGGGAGCCTTGTCGCGGAAACTTATCAACGG<br>GATAAGAGACAAGCAAAGTGGTAAAACTATTCTCGATTTTCTAAAGA<br>GCGACGGCTTCGCCAATAGGAACTTTATGGCCCTGATCCATGATGAC<br>TCTTTAACCTTCAAAGAGGATATACAAAAGGCACAGGTTTCCGGACA<br>AGGGGACTCATTGCACGAACATATTGCGAATCTTGCTGGTTCGCCAG<br>CCATCAAAAAGGGCATACTCCAGACAGTCAAAGTAGTGGATGAGCT

	AGTTAAGGTCATGGGACGTCACAAACCGGAAAACATTGTAATCGAG<br>ATGGCACGCGAAAATCAAACGACTCAGAAGGGGCAAAAAAACAGT<br>CGAGAGCGGATGAAGAGAATAGAAGAGGGTATTAAAGAACTGGGC<br>AGCCAGATCTTAAAGGAGCATCCTGTGGAAAATACCCAATTGCAGA<br>ACGAGAAACTTTACCTCTATTACCTACAAAATGGAAGGGACATGTAT<br>GTTGATCAGGAACTGGACATAAACCGTTTATCTGATTACGACGTCGA<br>TCACATTGTACCCCAATCCTTTTTGAAGGACGATTCAATCGACAATAA<br>AGTGCTTACACGCTCGGATAAGAACCGAGGGAAAAGTGACAATGTT<br>CCAAGCGAGGAAGTCGTAAAGAAAATGAAGAACTATTGGCGGCAGC<br>TCCTAAATGCGAAACTGATAACGCAAAGAAAGTTCGATAACTTAACT<br>AAAGCTGAGAGGGGTGGCTTGTCTGAACTTGACAAGGCCGGATTTA<br>TTAAACGTCAGCTCGTGGAAACCCGCGCCATCACAAAGCATGTTGC<br>GCAGATACTAGATTCCCGAATGAATACGAAATACGACGAGAACGATA<br>AGCTGATTCGGGAAGTCAAAGTAATCACTTTAAAGTCAAAATTGGTG<br>TCGGACTTCAGAAAGGATTTTCAATTCTATAAAGTTAGGGAGATAAA<br>TAACTACCACCATGCGCACGACGCTTATCTTAATGCCGTCGTAGGGA<br>CCGCACTCATTAAGAAATACCCGAAGCTAGAAAGTGAGTTTGTGTAT<br>GGTGATTACAAAGTTTATGACGTCCGTAAGATGATCGCGAAAAGCGA<br>ACAGGAGATAGGCAAGGCTACAGCCAAATACTTCTTTTATTCTAACA<br>TTATGAATTTCTTTAAGACGGAAATCACTCTGGCAAACGGAGAGATA<br>CGCAAACGACCTTTAATTGAAACCAATGGGGAGACAGGTGAAATCG<br>TATGGGATAAGGGCCGGGACTTCGCGACGGTGAGAAAAGTTTTGTC<br>CATGCCCCAAGTCAACATAGTAAAGAAAACTGAGGTGCAGACCGGA<br>GGGTTTTCAAAGGAATCGATTCTTCCAAAAAGGAATAGTGATAAGCT<br>CATCGCTCGTAAAAAGGACTGGGACCCGAAAAAGTACGGTGGCTTC<br>GATAGCCCTACAGTTGCCTATTCTGTCCTAGTAGTGGCAAAAGTTGA<br>GAAGGGAAAATCCAAGAAACTGAAGTCAGTCAAAGAATTATTGGGG<br>ATAACGATTATGGAGCGCTCGTCTTTTGAAAAGAACCCCATCGACTT<br>CCTTGAGGCGAAAGGTTACAAGGAAGTAAAAAAGGATCTCATAATT<br>AAACTACCAAAGTATAGTCTGTTTGAGTTAGAAAATGGCCGAAAAC<br>GGATGTTGGCTAGCGCCGGAGAGCTTCAAAAGGGGAACGAACTCGC<br>ACTACCGTCTAAATACGTGAATTTCCTGTATTTAGCGTCCCATTACGA<br>GAAGTTGAAAGGTTCACCTGAAGATAACGAACAGAAGCAACTTTTT<br>GTTGAGCAGCACAAACATTATCTCGACGAAATCATAGAGCAAATTTC<br>GGAATTCAGTAAGAGAGTCATCCTAGCTGATGCCAATCTGGACAAAG<br>TATTAAGCGCATACAACAAGCACAGGGATAAACCCATACGTGAGCAG<br>GCGGAAAATATTATCCATTTGTTTACTCTTACCAACCTCGGCGCTCCA<br>GCCGCATTCAAGTATTTTGACACAACGATAGATCGCAAACGATACAC<br>TTCTACCAAGGAGGTGCTAGACGCGACACTGATTCACCAATCCATCA<br>CGGGATTATATGAAACTCGGATAGATTTGTCACAGCTTGGGGGTGAC
5′UTR	GGACAGATCGCCTGGAGACGCCATCCACGCTGTTTTGACCTCCATAG<br>AAGACACCGGGACCGATCCAGCCTCCGCGGCCGGGAACGGTGCATT<br>GGAACGCGGATTCCCCGTGCCAAGAGTGACTCACCGTCCTTGACAC<br>G

T1-F-primer	CCAGGGTTTTCCCAGTCACGACGTTGTAAAACGAC
T2-F-primer	GGTGTTGGCGGGTGTCGGGGCTGGCTTAACTATGC
T3-F-primer	GAAGCATTTATCAGGGTTATTGTCTCATGAGCGGA
Gfp-template-R	TCAGTTGTACAGTTCATCCATGCCATGTGTAATCCC
SOX7-template-R	TCATGACACACTGTAGCTGTTGTAGTACGTGGCCGT
S-gene-template-R	TTATGTGTAATGTAATTTGACTCCTTTGAGCACTG
Cas9-template-R	TCACCCCCAAGCTGTGACAAATCTATCCGAGTTTC
Gfp-template-CTGT-R	ACAGTTGTACAGTTCATCCATGCCATGTGTAATCCC
Gfp-template-CTGC-R	GCAGTTGTACAGTTCATCCATGCCATGTGTAATCCC
Gfp-template-CTGG-R	CCAGTTGTACAGTTCATCCATGCCATGTGTAATCCC
Gfp-template-AAAA-R	TTTTTCAGTTGTACAGTTCATCCATGCCATGTGTAATCCC
Gfp-template-TTTT-R	AAAATCAGTTGTACAGTTCATCCATGCCATGTGTAATCCC
Gfp-template-CCCC-R	GGGGTCAGTTGTACAGTTCATCCATGCCATGTGTAATCCC
Gfp-template-GGGG-R	CCCCTCAGTTGTACAGTTCATCCATGCCATGTGTAATCCC
Gfp-template-1,2,3,4T-R	AAAATTGTACAGTTCATCCATGCCATGTGTAATCCC
Gfp-template-1A-R	TAAATTGTACAGTTCATCCATGCCATGTGTAATCCC
Gfp-template-1C-R	GAAATTGTACAGTTCATCCATGCCATGTGTAATCCC
Gfp-template-1G-R	CAAATTGTACAGTTCATCCATGCCATGTGTAATCCC
Gfp-template-2A-R	ATAATTGTACAGTTCATCCATGCCATGTGTAATCCC
Gfp-template-2C-R	AGAATTGTACAGTTCATCCATGCCATGTGTAATCCC
Gfp-template-2G-R	ACAATTGTACAGTTCATCCATGCCATGTGTAATCCC
Gfp-template-3A-R	AATATTGTACAGTTCATCCATGCCATGTGTAATCCC
Gfp-template-3C-R	AAGATTGTACAGTTCATCCATGCCATGTGTAATCCC

Gfp-template-3G-R	AACATTGTACAGTTCATCCATGCCATGTGTAATCCC
Gfp-template-4A-R	AAATTTGTACAGTTCATCCATGCCATGTGTAATCCC
Gfp-template-4C-R	AAAGTTGTACAGTTCATCCATGCCATGTGTAATCCC
Gfp-template-4G-R	AAACTTGTACAGTTCATCCATGCCATGTGTAATCCC
Gfp-NONpromoter-F	GGGAGACCCTCGAGGACAGATCGCCTGG
Gfp-NONpromoter-R	TTTTTTTCAGTTGTACAGTTCATCCATGCCATGTGT
DNA hairpin without promoter	6-FAM-GGGGTAATCATCAGCACTATAGGGAGACCCTCGAGGACAGATCAAAAAGATCTGTCCTCGAGGGTCTCCCTATAGTGCTGATGATTACCCC
DNA hairpin with a T7 promoter	6-FAM-AAAATAATACGACTCACTATAGGGAGACCCTCGAGGACAGATCAAAAAGATCTGTCCTCGAGGGTCTCCCTATAGTGAGTCGTATTATTTT
53-bp template	CTAATACGACTCACTATAGGGAGACCCTCGAGGACAGATCGCCTGGAGACGCC
RACE linker	GAUAAAAAGUAUUCUAUUGGUUUAGACAUCGGCACUAAUUCCGUUGGAUGGGCUGUCAUAACCGAUGAAUACAAAGUACCUUCAAAGAAAUUUAAGGUGUUGGGGAACACAGACCGUCAUUCGAUUAAAAGAAUCUUAUCGGUGCCCUCCUAUUCGAUAGUGGCGAAACGGCAGAGGCGACUCGCCUGAAACGAACCGC
3′RACE-gene-F	GCCACCTATACTTTCGCCAGCTGGCGTAATAGCGA
3′RACE-gene-R	CAGAGGCGACTCGCCTGAAACGAACCGCTATAGGT
3′RACE-plasmid-F	CGAACCGCTATAGGTGGCCCAATTAAGAATTCACT
3′RACE-plasmid-R	CTGGCGAAAGTATAGGTGGCCCAATTAAAAGCTTG
5′RACE-gene-F	ACCTATAGATAAAAAGTATTCTATTGGTTTAGAC
5′RACE-gene-R	ACCTATAATGGAAGCGTTCAACTAGCAGACCATTATCA
5′RACE-plasmid-F	CGCTTCCATTATAGGTGGCCCAATTAAGAATTCACTGG
5′RACE-plasmid-R	CTTTTTATCTATAGGTGGCCCAATTAAAAGCTTGGC

Table S2. 3′RACE results of antisense RNA.

	3′RACE	
	**Sequences**	**Reads/Total**
3′-terminal sequence of DNA template	ACACCCGCCAACACC	/
3′RACE results of antisense RNA	ACACCCGCCAACACC	1/10
	ACACCCGCCAA - - - -	1/10
	ACACCCGCCA - - - - -	1/10
	ACACCCGCCAACACCC	6/10
	ACACCCGCCAACACCCG	1/10

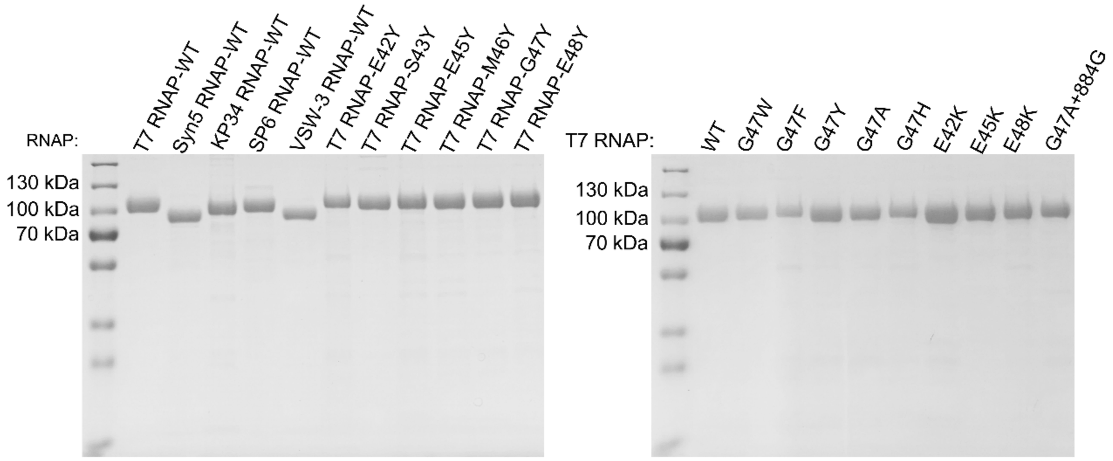

Figure S1. SDS-PAGE analysis of all the purified RNA polymerases and mutants in this study.

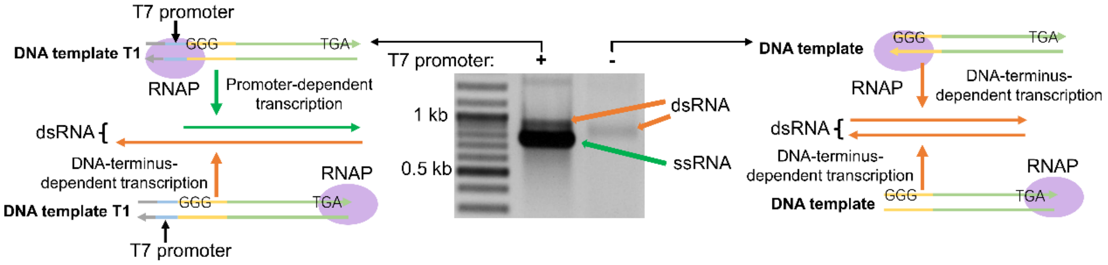

Figure S2. IVT by T7 RNAP on GFP DNA without T7 promoter. Agarose gel electrophoresis analysis of transcripts synthesized by wild-type T7 RNAP on GFP coding DNA with or without a T7 promoter. Gel bands corresponding to ssRNA and dsRNA are indicated by green and orange arrows, respectively. Schematics depicting the products formation are shown on both sides of the gel.
